# Resurgence of syphilis: focusing on emerging clinical strategies and preclinical models

**DOI:** 10.1186/s12967-023-04685-4

**Published:** 2023-12-18

**Authors:** Shun Xiong, Zhaoping Liu, Xiaohong Zhang, Shaobin Huang, Xuan Ding, Jie Zhou, Jiangchen Yao, Weiwei Li, Shuangquan Liu, Feijun Zhao

**Affiliations:** 1 Institute of Pathogenic Biology and Key Laboratory of Special Pathogen Prevention and Control of Hunan Province, Hengyang Medical College, University of South China, Hengyang, 421001 China; 2 Department of Clinical Laboratory Medicine, The First Affiliated Hospital, Institution of Microbiology and Infectious Diseases, Hengyang Medical College, University of South China, Hengyang, 421001 China

**Keywords:** Syphilis, *Treponema pallidum*, Diagnosis, Vaccine, Drug resistance, Models

## Abstract

Syphilis, a sexually transmitted disease (STD) caused by *Treponema pallidum* (*T. pallidum*), has had a worldwide resurgence in recent years and remains a public health threat. As such, there has been a great deal of research into clinical strategies for the disease, including diagnostic biomarkers and possible strategies for treatment and prevention. Although serological testing remains the predominant laboratory diagnostic method for syphilis, it is worth noting that investigations pertaining to the DNA of *T. pallidum*, non-coding RNAs (ncRNAs), chemokines, and metabolites in peripheral blood, cerebrospinal fluid, and other bodily fluids have the potential to offer novel perspectives on the diagnosis of syphilis. In addition, the global spread of antibiotic resistance, such as macrolides and tetracyclines, has posed significant challenges for the treatment of syphilis. Fortunately, there is still no evidence of penicillin resistance. Hence, penicillin is the recommended course of treatment for syphilis, whereas doxycycline, tetracycline, ceftriaxone, and amoxicillin are viable alternative options. In recent years, efforts to discover a vaccine for syphilis have been reignited with better knowledge of the repertoire of *T. pallidum* outer membrane proteins (OMPs), which are the most probable syphilis vaccine candidates. However, research on therapeutic interventions and vaccine development for human subjects is limited due to practical and ethical considerations. Thus, the preclinical model is ideal for conducting research, and it plays an important role in clinical transformation. Different preclinical models have recently emerged, such as in vitro culture and mouse models, which will lay a solid foundation for clinical treatment and prevention of syphilis. This review aims to provide a comprehensive summary of the most recent syphilis tactics, including detection, drug resistance treatments, vaccine development, and preclinical models in clinical practice.

## Introduction

Syphilis is a chronic multisystem disease caused by *Treponema pallidum* (*T. pallidum*), one of the oldest known diseases, with a resurgence in recent years. Since 2000, the prevalence of syphilis has increased significantly in developed countries. The Centers for Disease Control and Prevention (CDC) reported in 2017 that syphilis cases in the United States increased by up to 76% between 2013 and 2017 [1]. The World Health Organization (WHO) reports that there are about 6.3 million new cases of syphilis worldwide every year, and it is estimated that there will be 7 million new cases of syphilis in 2020 [1, 2]. Syphilis is known as “the great imitator” due to its variable clinical manifestations that can mimic other diseases. It not only causes chronic systemic multiple organ damage in adults, but also vertically spreads to the fetus through the placenta during pregnancy, leading to premature birth, miscarriage, stillbirth, and birth defects. The impact on sex with men (MSM), people living with HIV (PLWH), sex workers (SWs), and pregnant women is particularly serious [3]. Penicillin is still the preferred drug for the treatment of syphilis, but when it is not accessible (such as in allergic populations or countries where penicillin is not readily available), there are relatively few options for treating all phases of the disease. Although there have been numerous attempts to use alternative drugs in recent years, it is worth noting that resistance to alternative drugs in *T. pallidum* has now been found in several regions [1]. Therefore, we should pay attention to the resistance of *T. pallidum* in the screening of alternative drugs. Furthermore, it is worth noting that there is currently no clinical vaccine available for the prevention of syphilis. However, more study and knowledge of outer membrane proteins (OMPs) might reveal novel insights that could revolutionize the development of a syphilis vaccine.

Despite evidence-based curative treatment options with penicillin, it remains a public health threat with increasing prevalence over recent years. Research on therapeutic interventions and vaccine development for human subjects is limited due to practical and ethical considerations. Therefore, preclinical models are crucial for investigating syphilis pathogenesis and developing novel therapies and vaccines. Besides, clinical transformation is also greatly aided by preclinical models. Implementation of forward translation—the process of implementing basic research discoveries into practice—and reverse translation—the process of elucidating the mechanistic basis of clinical observations—practices could greatly enhance our ability to develop effective anti-syphilis strategies. The aim of this review is to integrate the extensive literature to gain new insights and optimize current protocols for syphilis diagnosis, treatment, and prevention, as well as preclinical models.

## Diagnosis

### Serologic diagnosis

Currently, serological testing is the most mainstream laboratory diagnosis method for syphilis, mainly including the *nontreponema test* [rapid plasma reagin (RPR) test or venereal disease research laboratory (VDRL) test] and the *treponema test* [*Treponema pallidum* particle agglutination assay (TPPA), various enzyme immunoassays (EIAs), chemiluminescence immunoassays (CIAs) and immunoblots, or rapid treponemal, et al.] [4]. Non-treponemal rapid plasma regain (RPR) flocculation tests are used to assess disease activity, assess response to treatment, and diagnose reinfection or recurrence. However, non-treponemal testing is for antibodies against lipoidal antigens, which are non-specific and usually not detected until a few weeks after infection. Treponema testing is more sensitive to early infection, and treponema serology is often used to detect treponema IgG (CLIA) and TPPA) to investigate possible cases of syphilis. Treponema tests target treponema pallidum-specific proteins, and many current commercial tests mainly use *T. pallidum* antigens (Tp15, Tp17, and Tp47) to detect IgM, IgG, or both. Here we summarize a large number of serological diagnostic candidate antigen studies [5–15] (Table 1). Besides, the response intensity and rate of antibody production to these candidate antigens may also serve as sensitive indicators for the early diagnosis of syphilis. Despite the fact that these antigens are useful in the serological diagnosis of syphilis, treponeme-specific diagnostics such as enzyme-linked immunosorbent assay (ELISA) are unable to evaluate syphilis therapy. Notably, Zhao discovered a highly significant positive association between the difference in A450 nm values for Tp0971 and the RPR titre change before and after syphilis treatment, indicating the potential of Tp0971 in the assessment of the effectiveness of syphilis therapies [9].

**Table 1:** Studies on candidate antigens of *T. pallidum* for serological diagnosis

Gene (ORF number)	Protein name	Protein description	Immunoreactivity with syphilis sera (ELISA test ratio)^a^	Immunoreactivity with syphilis sera (2DGE immunoblot)^b^	Seroreactivity at syphilis stages	Sensitivity/specificity (%)	detection method	Published (year)	References
Inner membrane lipoproteins									
*tp0171*	Tp15	15 kDa lipoprotein	None	+++	All stages	100/100	Western blot	2001	[14]
*tp0435*	Tp17	17 kDa lipoprotein	9.6–16.6	+++	All stages	96/100	western blot	2001	[14]
*tp0574*	Tp47	47 kDa penicillin- binding protein, carboxypeptidase	2.9–10.0	+++	All stages primary and secondary syphilis	100/20 79.8/95.3	western blot PCR	2001 2015	[14] [16]
*tp0768*	TmpA	44.5 kDa lipoprotein	8.2–15.3	+++	All stages	76–100/99.6	ELISA/western blot	1989/2001	[14, 15]
*tp0319*	TmpC	35 kDa lipoprotein, purine nucleoside receptor A lipoprotein	2.8–6.2	+/++	All stages	100/100	–	1996	[108]
*tp0684*	Tp38	38 kDa lipoprotein, methylgalactoside ABC transporter, galactose/glucose-binding lipoprotein	6.8–19.0	+++	All stages	–	ELISA	2004	[109]
*tp0821*	Tp32	32 kDa lipoprotein, l-methionine-binding lipoprotein	1.0–1.7	None	All stages	91.0–98.3/94.3–100	ELISA	2016	[12]
Surface-exposed and outer membrane associated proteins									
*tp0897*	TprK	Heterogenic antigen variable by gene conversion	None	None	–	–	ELISA	2004/2014	[110–112]
*tp0663*	TROMP-2	28-kDa outer membrane protein, FlaA homolog	0.8–1.9	None	All stages	98.83/100	ELISA	2016	[10]
*tp0326*	Tp92	BamA (*β*-barrel assembly machinery protein A) ortholog	1.2–2.6	None	Mostly at primary stage; lower reactivity in secondary and early latent stage	86/99 98/97	ELISA	2013/2003	[6, 7]
*tp0453*	Tp0453	Proposed carrier of lipids and glycolipids	None	+/++	–	98/100 100/100	ELISA	2013/2003	[6, 7]
Adhesins									
*tp0155*	Tp0155	Binds to the matrix form of fibronectin and exhibit peptidase enzymatic activity	None	None	low reactive at primary stage	–/27.9	ELISA	2003	[7]
*tp0483*	Tp0483	Binds to both the soluble and matrix forms of fibronectin	None	None	Low reactive at primary stage	–/41.8	ELISA	2003	[7]
*tp0751*	Tp0751	25.8 kDa protein; it binds to laminin and exhibits metalloprotease activity	None	None	–	–/41.8	ELISA	2003	[7]
Putative periplasmic proteins									
*tp0257*	Gpd	Glycerophosphodiester phosphodiesterase, binds Fc-fragment of human IgA, IgD, and IgG immunoglobulins	3.0–7.3	None	All stages	91/93	ELISA	2003	[7]
*tp1038*	TpF1	Bacterioferritin, homodecamer from 19-kDa subunits	0.8–2.2	+++	All stages	93–100/100	ELISA	2013	[8]
Flagellar proteins									
*tp0868*	FlaB1	Flagellar filament 34.5-kDa core protein	None	+++	All stages	95.4/98.9	ELISA	1993	[13]
*tp0792*	FlaB2	Flagellar filament 33-kDa core protein	None	+++	All stages	92.6/95.8	ELISA	1993	[13]
*tp0870*	FlaB3	Flagellar filament 31-kDa core protein	None	+++	All stages	95.1/95.8	ELISA	1993	[13]

*ELISA* enzyme-linked immunosorbent assay, *None* unknown, *2DGE* two-dimensional gel electrophoresis

^a^The chemiluminescence ratio refers to the relative light units resulting from the binding of serum Ig to the *T. pallidum*-GST fusion protein, divided by the value obtained when wells were coated with GST alone. Significant reactivity is indicated by boldface

^b^Reactivity was evaluated subjectively as nonreactive (−), weakly reactive (+), moderately reactive (++), or highly reactive (+++)

### Diagnosis by nucleic acid amplification test (NAAT)

**Fig. 1:**
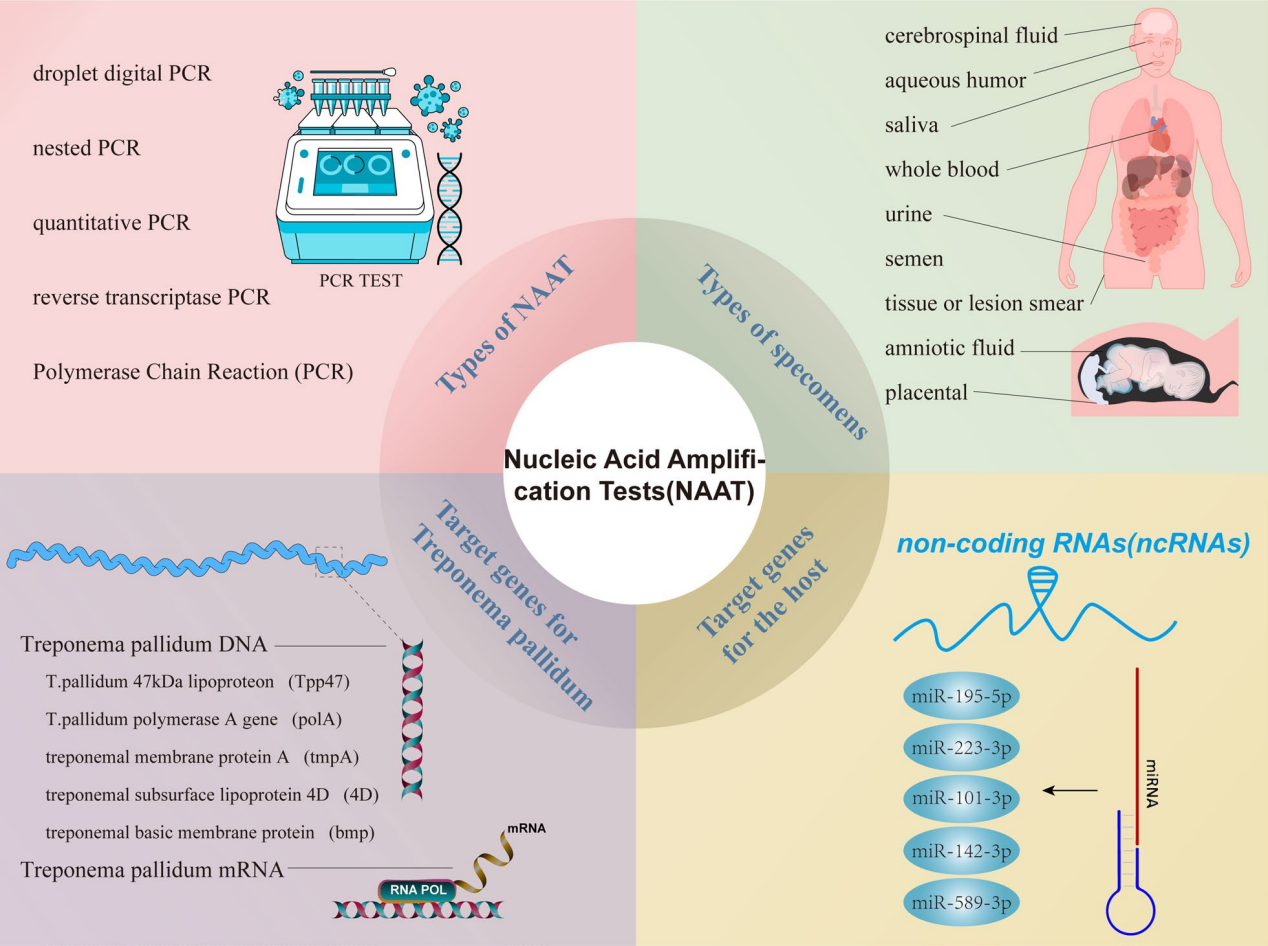
Diagnosis by nucleic acid amplification test (NAAT). Molecular assays with nucleic acid amplification tests (NAAT) are used for direct detection to improve diagnostic sensitivity. Types of NAAT include polymerase chain reaction (PCR), nested PCR, quantitative PCR, reverse transcriptase PCR, and droplet digital PCR (ddPCR). Notably, nPCR has a higher specificity and sensitivity than conventional PCR, especially in seronegative and serologically different individuals. In addition to blood and cerebrospinal fluid (CSF), scholars have begun to explore other types of specimens, such as tissue or lesional smears, saliva, urine, aqueous humor, semen, amniotic fluid, and placental tissue. NAAT mainly aims at three target genes of *T.pallidum*, including the DNA polymerase I gene (polA), Tp47(tp0574), and bmp; besides, 16S rRNA, tmpC, subsurface lipoprotein 4D (4D), and tmpA were involved. *T. pallidum* can be diagnosed in part by targeting its DNA, but mRNA or non-coding RNAs (ncRNAs) can also be targeted for complementary diagnosis

In particular, NAAT has gained popularity for diagnosing infectious disorders caused by organisms that are difficult to culture. Researchers have been increasingly turning to NAAT as a means of detecting *T. pallidum* DNA in a wide range of sample types and disease states. Figure 1 graphically depicts the use of the nucleic acid amplification test in the diagnosis of syphilis. NAAT mainly aims at three target genes of *T. pallidum*, including the DNA polymerase I gene (*polA*), *Tp47*(*tp0574*), and *bmp* [16–18]; besides, 16S rRNA, *tmpC*, and *tmpA* were involved [19, 20]. Some studies showed that five types of NAAT, including routine PCR, real-time PCR (qPCR), reverse transcription PCR (RT-PCR), nested PCR (nPCR), droplet digital PCR (ddPCR), and loop-mediated isothermal amplification (LAMP) assays could be used to diagnose syphilis [17, 21–25]. In parallel, a recent study developed assays that pair PCR pre-amplification of the *tp0574* gene of *T. pallidum* with CRISPR-LwCas13a, which outperformed *tp0574* real-time PCR and rabbit-infectivity testing in terms of sensitivity and specificity [26]. In addition to blood and CSF, scholars have begun to detect *T. pallidum* DNA in other types of specimens, such as saliva, atrial fluid (aqueous humor), urine, semen, oropharynx, and anorectum, during early syphilis stages as a proxy for transmissibility [25, 27–30]. It's important to note that nPCR has far greater specificity and sensitivity than traditional PCR, notably in seronegative individuals and those with discrepant serology [24]. In a recent study, researchers found that *T. pallidum* DNA could be identified in saliva at all syphilis stages, with greater detection rates in saliva than in plasma, with the exception of primary syphilis [27]. Saliva samples may be a sensitive diagnostic fluid for syphilis, and they also have the benefits of being convenient and non-invasive, making them a viable approach for monitoring *T. pallidum* DNA elimination as an indication of therapy efficacy [27]. Additionally, *T. pallidum* DNA in urine was detectable in individuals throughout the spectrum of syphilis severity, and loads were larger in urine sediment than in urine supernatant. Patients with both primary and secondary syphilis have a high chance of detection. Obtaining a large quantity of *T. pallidum* DNA from urine may be a good idea due to the sample’s abundance and the ease with which it may be collected [25].

*T. pallidum* can be diagnosed in part by targeting its DNA, but mRNA or non-coding RNAs (ncRNAs) can also be targeted for complementary diagnosis. Infection with *T. pallidum* may induce tissue damage, and microRNAs have a regulatory function in the immune response to *T. pallidum* infection. Recent research has demonstrated that *T. pallidum* infection increases the expression of miR-101-3p, inhibiting the TLR2 signaling pathway and resulting in decreased cytokine production [31]. Furthermore, miRNA expression differed in peripheral blood mononuclear cells (PBMCs) at various phases of *T. pallidum* infection [32]. As a result, the microRNAs in PBMCs might singly or jointly be potential diagnostic biomarkers at different stages of syphilis [31–35]. MiR-195-5p, miR-101-3p, and miR-223-3p alone or in combination can specifically distinguish syphilis patients from non-syphilis patients [32, 33]; miR-101-3p can be used as a diagnostic biomarker for patients with primary syphilis [31]. Additional research has indicated that miR-142-3p is a promising PBMC-based specific biomarker for secondary syphilis [34]. In addition, miR-338-5p and miR-101-3p can be used as diagnostic indicators of serofast state [31, 35]. Interestingly, miR-195-5p, miR-223-3p, and miR-589-3p showed significant differences in the diagnosis of serofast and serologically cured states [32]. MicroRNAs may be employed as a non-invasive biomarker of *T. pallidum* infection to aid in the diagnosis of the disease. However, further research is required before clinical applications may be realized.

The most often used non-treponemal test for neurosyphilis is CSF-VDRL. A positive CSF-VDRL test is regarded neurosyphilis diagnostic, while a negative result does not rule out the diagnosis. Numerous studies have shown that high concentrations of CXCL13 in the CSF may be possible biomarkers of neurosyphilis, especially for asymptomatic neurosyphilis, adding to the growing list of diagnostic molecular markers for syphilis. CXCL13 has other interesting applications, including treatment monitoring in neurosyphilis [36]. Elevated concentrations of CXCL8, CXCL10, and IL-10 may also be potential biological markers of neurosyphilis, especially asymptomatic neurosyphilis [37–39]. Meanwhile, the increased levels of CXCL9, CXCL7, CCL24, IL-17, IL-26, and migration inhibitory factor (MIF) of macrophages in the CSF of neurosyphilis patients suggested their role as promising differential diagnostic tools for neurosyphilis [37, 40–42]. Although these cytokine changes are still in their infancy for the diagnosis of neurosyphilis, their importance cannot be ignored due to the lack of ideal neurosyphilis biomarkers.

### Histological diagnosis

For almost a decade, modern mass spectrometry has been used in the biological study of human metabolites and proteins (proteomics and metabolomics, respectively). However, the use of mass spectrometry to diagnose syphilis is still in its infancy. High-sensitivity proteomics, which relies on mass spectrometry (MS) to identify proteins, has, however, been a major driving force. In recent years, an MS-based approach has been successfully applied in numerous clinical microbial protein screening studies. In the past decades, researchers have also used pre-MS analysis based on gel technology to identify *T. pallidum* protein peptides. In 2016, McGill first studied the purified *T. pallidum* proteome using matrix-assisted laser desorption /ionization time of flight (MALDI-TOF/TOF) and electrospray ionization (ESI-LTQ-Orbitrap), which identified 557 unique *T. pallidum* proteins [43]. Subsequently, two different MS-based proteomics approaches were used to analyze *T. pallidum* proteins in urine samples from syphilis patients, yielding the identification of 26 peptides corresponding to four *T. pallidum* proteins [44]. Interestingly, identification of *T. pallidum*-specific proteins in sera of syphilis patients based on liquid chromatography coupled with tandem mass spectrometry (LC–MS/MS) also revealed high expression levels and low homology of Tp0369 [45], strongly suggesting that Tp0369 is a promising candidate peptide target for syphilis early diagnosis, so as to overcome the nonspecific problem of antigen detection [44, 45].

Global metabolomics analysis can provide substantial information on possible diagnostic biomarkers for pathogens. The metabolite profile of cerebrospinal fluid (CSF) from neurosyphilis patients determined by untargeted metabolomic analysis showed significant differences in d-mannose, l-gulono-gamma-lactone, *S*-methyl-5ʹ-thioadenosine, hypoxanthine, and *N*-acetyl-l-tyrosine, with the largest difference in *N*-acetyl-l-tyrosine by the student’s t test [46]. In addition, LC–MS revealed that the levels of bilirubin, l-histidine, prostaglandin E2, alpha-kamlolenic acid, butyryl-l-carnitine, and palmitoyl-l-carnitine were significantly reduced in the CSF of neurosyphilis patients, suggesting them to be novel potential biomarkers of neurosyphilis [47]. Untargeted metabolomic analysis of neurosyphilis patient serum revealed that several metabolites, including trimethylamine *N*-oxide, l-arginine, lysoPC (18:0), betaine, and acetylcarnitine, were significantly higher in syphilis patients than in healthy controls, with trimethylamine *N*-oxide being the best candidate metabolic biomarker to differentiate the sera of syphilis patients and healthy controls [48]. A rise in oxidative stress products (AOPP, carbonyls) and nitrosative stress markers (nitrates/nitrites) in the sera of syphilis patients has been observed since the disease's earliest stages. These differential metabolites, which could potentially improve neurosyphilis and syphilis diagnostics in the future, deserve further exploration.

### Others in diagnosis

Syphilis can be diagnosed by a number of different methods, such as those listed above as well as morphological observation, immunohistochemistry (IHC), the rabbit infectivity test (RIT), and in vitro culture. Dark-field microscopy (DFM) or direct fluorescent antibody (DFA) testing is the primary method of morphological observation [4]. This allows for the direct identification of spirochetes with characteristic shapes and movements from lesion exudate. When serologic tests fail to find T. pallidum antibodies, immunohistochemistry (IHC) might be used as a supplement. Although the IHC approach has high specificity for secondary syphilis, its sensitivity varies from 49 to 92% [49]. Furthermore, immunohistochemistry requires specific equipment and stains, might cross-react with different spirochetes, and yields subjective results [50]. *T. pallidum* could also be detected in tissue from mucocutaneous syphilis lesions at all stages using a combination of focus-floating microscopy (FFM) and polymerase chain reaction (PCR), which is a speedy, reliable, economical, and enhanced immunohistochemical technique [51]. The New Zealand White (NZW) rabbit has long been recognized as the most useful practical animal model for determining in vivo infectivity of *T. pallidum* [51], and for good reason. While RIT was formerly considered a gold standard for the sensitive detection of *T. pallidum* in clinical samples, it is no longer used as such and is instead used as a benchmark against which the sensitivity of more modern techniques like PCR is evaluated. *T. pallidum* has been cultured in vitro for extended periods of time using a technique based on TpCM-2 media and the Sf1Ep co-culture system [52]. To effectively identify and diagnose syphilis, this method will need more development, although it shows great promise.

## Antibiotics and treatment regimens

For a long time, benzathine penicillin G (BPG), administered by injection, has been the preferred drug for the treatment of patients at all stages of syphilis. The preparation used (i.e., benzathine, aqueous procaine, or aqueous crystalline), dosage, and length of treatment depend on the stage and clinical manifestations of the disease. A single dose of long-acting benzathine penicillin G of 2.4 million units, each side of the intramuscular injection of 1.2 million units, is an effective treatment for early stage syphilis (primary and secondary syphilis and early latent syphilis), whereas 2.4 million units administered intramuscularly weekly for 3 consecutive weeks is recommended for late latent syphilis and tertiary syphilis. Some experts recommend that primary, secondary, and early latent cases be treated with two doses of long-acting benzathine penicillin G 2·4 million units one week apart, particularly in the third trimester [53]. Short-acting penicillin agents are not adequate to cure syphilis. Table 2 details the recommended and alternative syphilis treatment regimens from the Centers for Disease Control and Prevention (CDC). Notably, HIV-positive patients with early syphilis are more likely to have cerebrospinal fluid abnormalities than HIV-negative patients, so all people infected with HIV and syphilis should undergo careful neurologic ocular and otic examination tests [54]. However, HIV status does not affect the CDC treatment recommendations for all stages and for neurosyphilis, ocular, and otic syphilis. People with HIV and neurosyphilis should be treated according to the recommendations for persons with neurosyphilis and without HIV infection. Besides, available data suggest no clinical benefit to multiple doses of benzathine penicillin G for early syphilis in HIV-positive patients [55]. Interestingly, in HIV-infected people with early syphilis, a single dose of BPG plus doxycycline achieved a better serologic response than a single dose of BPG [56]. In addition, intravenous penicillin G is the only documented effective treatment for syphilis in pregnancy, and penicillin G is also the only known effective antibacterial agent for the treatment of fetal infection and the prevention of congenital syphilis. Pregnant women with syphilis at any stage who report a penicillin allergy should be desensitized and treated with penicillin. The treatment of congenital syphilis and neurological syphilis will not be discussed in this section due to their complexity.

**Table 2:** Recommended and alternative syphilis treatment regimens [130]

	Recommended regimen	Alternative regimen 1	Alternative regimen 2
Primary and secondary syphilis			
In nonpregnant adults, including adults with HIV	Benzathine penicillin G 2.4 million units IM in a single dose	Doxycycline, 100 mg orally twice a day for 14 days Tetracycline, 500 mg orally 4 times a day for 14 days	Ceftriaxone, 1 g daily, IM or IV, for 10–14 days
In pregnancy	Penicillin G benzathine, 2·4 million units in a single intramuscular dose	Those allergic to penicillin should be desensitized and treated with penicillin G	
Among infants and children	Benzathine penicillin G 50,000 units/kg body weight IM, up to the adult dose of 2.4 million units in a single dose		
Early latent syphilis			
In non-pregnant adults, including adults with HIV	Benzathine penicillin G 2.4 million units IM in a single dose		
In pregnancy	Benzathine penicillin G 2.4 million units IM in a single dose	Those allergic to penicillin should be desensitized and treated with penicillin G	
Late latent syphilis			
In non-pregnant adults, including adults with HIV	Benzathine penicillin G 7.2 million units total, administered as 3 doses of 2.4 million units IM each at 1-week intervals	Doxycycline, 100 mg orally twice a day for 28 days Tetracycline, 500 mg orally 4 times a day for 28 days	Ceftriaxone may be effective; but optimal dose and duration are unknown
In pregnancy	Benzathine penicillin G 7.2 million units total, administered as 3 doses of 2.4 million units IM each at 1-week intervals	Those allergic to penicillin should be desensitized and treated with penicillin G	
Tertiary syphilis			
With gummas and cardiovascular manifestations but not neurosyphilis	Benzathine penicillin G 7.2 million units total, administered as 3 doses of 2.4 million units IM each at 1-week intervals		
Neurosyphilis, ocular syphilis, and otosyphilis			
Including adults with HIV	Aqueous crystalline penicillin G 18–24 million units per day, administered as 3–4 million units IV every 4 h or continuous infusion for 10–14 days	Procaine penicillin G 2.4 million units IM once daily plus Probenecid 500 mg orally 4 times a day, both for 10–14 days	Benzathine penicillin, 2.4 million units IM once per week for 1–3 weeks, can be considered after completion of these neurosyphilis treatment regimens
Congenital syphilis			
Confirmed, highly probable, or possible congenital syphilis	Aqueous crystalline penicillin G 100,000–150,000 units/kg/body weight/day, administered as 50,000 units/kg body weight/dose IV every 12 h during the first 7 days of life and every 8 h thereafter for a total of 10 days	Procaine penicillin G 50,000 units/kg body weight/dose IM in a single daily dose for 10 days	Benzathine penicillin G 50,000 units/kg body weight/dose IM in a single dose
Among infants and children	Aqueous crystalline penicillin G 200,000–300,000 units/kg body weight/day IV, administered as 50,000 units/kg body weight every 4–6 h for 10 days		
Management of sex partners			
Persons who have had sexual contact with a person who receives a diagnosis of primary, secondary, or early latent syphilis < 90 days	Should be treated presumptively for early syphilis		
Persons who have had sexual contact with a person who receives a diagnosis of primary, secondary, or early latent syphilis > 90 days	Should be treated presumptively for early syphilis or treated empirically		

IM, intramuscularly; IV, intravenous

Effective non-penicillin-based regimens are required in patients with penicillin allergies, would provide alternative treatments during shortages of penicillin, and might be more conducive to administration and outpatient management. Doxycycline, tetracycline, and ceftriaxone can be used as substitutes for people who cannot use penicillin. The clinical and serologic outcomes of oral doxycycline treatment are similar to those of penicillin-based therapy, but a randomized controlled trial is necessary to determine the effectiveness of doxycycline in the treatment of early neurosyphilis. There have been other trials showing that doxycycline is effective in treating syphilis and syphilitic uveitis in pregnant women who were unable to undergo a penicillin desensitization [57, 58]. Additionally, there is increasing interest in using doxycycline for prophylaxis of this infection. *T. pallidum* showed a significant level of susceptibility to doxycycline in vitro, and post-exposure prophylaxis (PEP) with doxycycline has been shown to be effective in preventing syphilis infection [59, 60]. Since tetracycline has more frequent dose requirements and more potential for gastrointestinal adverse effects, compliance is probably better with doxycycline than tetracycline [61]. Additionally, in both latent and primary syphilis patients, the ceftriaxone regimen has been shown to be noninferior to the BPG regimen. Syphilitic uveitis, neurosyphilis, and ocular syphilis, as well as syphilis-related membranous nephropathy, may respond well to ceftriaxone treatment in the absence of penicillin G [62–65]. However, the data are insufficient to recommend ceftriaxone or other cephalosporins for treatment of maternal infection and prevention of congenital syphilis [66]. More caution is needed when administering ceftriaxone to patients who are also allergic to penicillin due to the possibility of cross-allergy. The effectiveness, ideal dosage, and duration of amoxicillin in patients with various stages of syphilis must still be determined by additional research.

It is worth mentioning that, while researchers continue to investigate new syphilis treatment options, the emergence of worldwide antibiotic resistance requires us to pay attention. The genomic epidemiology of syphilis has revealed the independent emergence of macrolide resistance in several circulating lineages [67, 68]. According to research published in the 23S rRNA gene of *T. pallidum*, both A2058G and A2059G mutations are associated with failure of macrolide treatment [69]. These mutations are widespread throughout the world [70]. In addition, BPG was effective in NZW rabbits infected with strains harboring 23S rDNA mutations, but azithromycin failed [71]. Consequently, azithromycin cannot be used as an alternate therapy for syphilis patients at this time, despite the advice of recommendations [70]. In comparison to A2059G, the mutation A2058G confers macrolide resistance more commonly. Nevertheless, no strains with both mutations have been reported to date. In addition, the acquired tetracycline resistance gene tetB was amplified from the total DNA of a reliable number of *T. pallidum*-positive samples (i.e., 15/171) collected between 2014 and 2015 in Shandong province, and no point mutations in the 16SrRNA gene were detected. Tetracyclines have been proposed as an alternative to BPG for the treatment of syphilis; nevertheless, there is concern about the potential emergence of tetracycline-resistant strains of *T. pallidum*. Table 3 summarizes in detail the mutation sites associated with drug resistance in *T. pallidum* strains. With the emergence and spread of resistant *T. pallidum*, the availability of treatment options is decreasing. Fortunately, there is still no evidence of penicillin resistance. Hence, penicillin is the recommended course of treatment for syphilis.

**Table 3:** Mutant loci associated with drug resistance of *T. pallidum* strains

Resistance type	Resistance gene	Mutation position	Country/city	Mutation rates (%)	Collection year	References
Macrolide resistance	23S rRNA	A2058G	Hunan, China	97.5	2013–2015	[113]
		A2058G	Guangxi Zhuang Autonomous Region, China	91.0	2012–2014	[114]
		A2058G	Xiamen, China	100	2016–2017	[115]
		A2058G	Cuba	61	2012–2015	[116]
		A2058G	Sydney, Australia	84	2004–2011	[117]
		A2058G	Czech	86.7	2004–2017	[118]
		A2058G	Tuva Republic, Russia	2.4	2013–2014	[119]
		A2058G	Buenos Aires, Argentina	9.5	2006–2013	[120]
		A2058G	Manitoba, Canada	97.3	2012–2016	[121]
		A2058G	Northern Italy	92.5	2016–2017	[122]
		A2058G	Japan	83	2017	[123]
		A2058G	Barcelona, Spain	99.1	2015	[124]
		A2058G	France	85	2012–2017	[125]
		A2058G	Brazilian Marajó Archipelago	14.8	2018–2019	[126]
		A2058G	Southern Africa	23	2008–2018	[127]
		A2059G	Czech	3.3	2004–2017	[118]
		A2059G	Buenos Aires, Argentina	4.8	2006–2013	[120]
		A2059G	Northern Italy	1.9	2016–2017	[122]
		A2059G	Manitoba, Canada	2.7	2012–2016	[121]
		A2059G	Barcelona, Spain	0.9	2015	[124]
		A2059G	Brazilian Marajó Archipelago	16.2	2018–2019	[126]
Tetracycline resistan**ce**	tetB	–	Shandong, China	8.8	2014–2015	[128]

## Vaccines

Research on a vaccine against *T. pallidum* has moved at a slower pace than that into vaccines against other diseases. *T. pallidum*’s outer membrane is fragile, in vitro mass growth of *T. pallidum* is challenging, and the mature use of transgenic procedures all limit the relevant technology, making further advances in this area necessary. Vaccines against *T. pallidum* are currently available in a variety of forms, including live attenuated, inactivated, DNA, and recombinant proteins (Table 4).

**Table 4:** Studies on antigens for *T.pallidum* vaccines

Vaccine type	TP/rTP protein	Inactivation metho/characteristics	Adjuvant	Immunization dosage and route	Ulcer rate (%)	Immunization effect	References
Inactivated vaccines	*T. pallidum*	Stored at 4 ℃, for from 7 to 10 days	None	8 × 10^9^/Iv	0	Part of the samples were positive for lymph node metastasis	[72]
	*T. pallidum*	Deal with penicillin added stored at 37℃ for 24 h and at 4 °C for an additional 6 to 9 days	None	8 × 10^9^/Iv	0.20	Partial	[72]
	*T. pallidum*	Ultraviolet light	None	3.7 × 10^9^/Iv	0	Complete	[129]
DNA vaccine	pcDNA3/FlaB3	–	None	3 × 150 μg/Im	20	Partial	[76]
	Tp92	OMP, highly conserved, highly homologous	None IL-2 IL-2 + CS CS	3 × 100 μg/Im	30 10 7.5 25	Partial	[74]
	Gpd	Lipoprotein, binds to the opsonin antibody, correlated with immune escape	None IL-2 CS Cs + IL-2 CpG + IL-2	3 × 100 μg/Sc 100 μg Gpd-IL-2/Im + Gpd-IL-2 + CpG/Nasal	37.5 10 40 4.17 8.3	Partial	[75]
Subunit vaccine	FlaB3	Associated with pathogen diffusion	Freund’s	3 × 150 μg/Im	14.29	Partial	[76]
	Tp0136	OMP, lipoprotein, adhesion protein	Freund’s	3 × 150 μg/Sc	12.5	Partial	[79]
	Tp0136	OMP, lipoprotein, adhesion protein	Titer Max Gold	500 μg + 3 × 250 μg/Sc + Im	92.5	No protection	[90]
	Tp0126	OMP (OmpW family)	mSAS	5 × 250 μg/Id, Im, sc	100	No protection	[92]
	Tp0663	OMP	Freund’s	3 × 150 μg/Sc	0	Partial	[79]
	Tp0715	Adhesive, associated with pathogen Colonization	TiterMax Gold	4 × 5.2 μg/Sc + 2 × 13 μg/Iv	47	Complete	[81]
Heterologous Expression in *Borrelia burgdorferi*	TprK	OMP, undergoes antigenic variation at 7 variable regions, and variants are selected by immune pressure	None	10^10^/Im	44	Partial	[82]
	Tp0435	Lipoprotein, a periplasmic antigen that was also shown on the pathogen surface	None	10^10^/Im	100	No protection	[82]

rTP protein, recombinant *Treponema pallidum* protein; OMP, outer membrane protein; Partial, partial protection

### Inactivated and live attenuated vaccines

Metzger first demonstrated that *T. pallidum* could produce partial protection by immunizing NZW rabbits with *T. pallidum* stored at 4 °C, heated at 100 °C, or treated with penicillin by either intravenous or intradermal injections[72]. Miller claims that inoculating NZW rabbits with a radioactively inactivated form of *T. pallidum* provided them with protection against infection for a year. In clinical trials, this vaccine has been demonstrated to be more effective than any other in preventing the disease. One of the limitations of inactivated vaccines is that large quantities of *T. pallidum* are not available for use. An improved in vitro cell co-culture method and the first attempt at genetic engineering of *T. pallidum* have opened up many possibilities, including providing *T. pallidum* for inactivated vaccines and possibly even targeting virulence factors responsible for immune escape and persistence to obtain attenuated strains to inform vaccine development efforts [52, 73]. However, difficulties in producing large-scale in vitro cultures have impeded the future development of inactivated and live-attenuated vaccines.

### DNA vaccines

As mentioned previously, Tp92 + IL-2, Gpd + IL-2, and FlaB3 immunized NZW rabbits displayed attenuated lesions as well as significant decreases in blood, liver, spleen, and testis *T. pallidum* levels [74–76]. It should be noted, however, that DNA vaccines are at risk of insertional mutagenesis, which occurs when exogenous genes are incorporated into the chromosomes of host cells and can lead to immunological tolerance after several application sessions.

### Recombinant protein vaccines

Vaccines made from recombinant proteins have been the subject of much research and testing, since they are free of potentially dangerous active components and can be mass-produced at a low price. The first step in creating a *T. pallidum* protein vaccine is to screen and investigate *T. pallidum* proteins that interact with host cells during the first stages of infection. Growing evidence suggests that *T. pallidum*’s early adherence and colonization of target cells in hosts is crucial to the establishment of its eventual syphilis, which is connected with immune escape and immunological tolerance in the chronic systemic infection of syphilis. Thus, the outer membrane proteins and various membrane lipoproteins of the *T. pallidum* that are the earliest to come into direct contact with host cells have become the focus of research on *T. pallidum* vaccines[77]. In particular, rare outer membrane proteins of *T. pallidum* (e.g., Tp92) and adhesins that bind to extracellular matrix proteins (e.g., Tp0751 and Tp0136). Besides, numerous studies have also confirmed that the outer membrane proteins Tp92, Tp0769, Tp0663, and TprK; and the outer membrane lipoproteins Tp0751 and Tp0136 can all induce partial protective immunity [78–81].

Only Tp92 of *T. pallidum* has sequence similarity with gram-negative outer membrane proteins (OMPs). There is a considerable degree of homology between the amino acid sequences of Tp92 from 11 different strains representing 4 different pathogenic treponemes, and vaccination with recombinant Tp92 provides some immunological protection for NZW rabbits against *T. pallidum* infection [78]. TprK (Tp0897) is a target of opsonic antibodies and the protective immune response since it is one of *T. pallidum*’s rare outer membrane proteins (REMP). *T. pallidum* relies heavily on antigenic variation to evade the immune system, and the TprK variable regions have been shown to be an important target of the humoral immune response during experimental infection. A recent study found that immunization with the recombinant protein TprK dramatically reduced the frequency of lesions, sped up the healing process, and slowed the progression of cutaneous lesions from early stages to ulceration [80, 82]. In addition, Nikhat Parveen found that immunizing NZW rabbits with non-pathogenic B. burgdorferi expressing TprK resulted in a robust humoral response and partial protection [82]. It is important to remember that TprK displays a great deal of variation both across and within strains. This gene’s sequence diversity is partitioned into seven distinct variable (V) regions (V1–V7) that are separated by conserved sequences. Additional genomic sequencing showed that individuals with primary and secondary syphilis had substantial variability at the intrastrain level in V6 [83]. Meanwhile, rabbits pre-immunized with V6 region synthetic peptides had a more rapid accumulation of V6 variant treponemes than control rabbits [84]. Furthermore, the amino acid sequences of V6 also presented increased diversity at the interstrain level over time during *T. pallidum* infection [85]. Consequently, V6 may be the initial region to alter in primary syphilis samples. Recent genome sequencing has shown even more variation in genes like TprK that are candidates for vaccines, laying the groundwork for vaccine development [86]. Besides, the homologous protein family of Tpr, including TprL, TprC, and TprD, also has potential for vaccine development [87, 88]. Immunization of guinea pigs with Tp0769 (also known as TmpB) induces protection against *T. pallidum* infection [89]. In addition, NZW rabbits immunized by Tp*01*36 exhibited increased specific antibody titers, attenuated lesion development, increased cellular infiltration at the lesion sites, and inhibition of treponemal dissemination to distant organs compared to the unimmunized animals [79, 90]. Popliteal lymph nodes were transplanted from killed rabbits with healed syphilitic lesions; however, DFA showed the presence of *T. pallidum* in the testicles of all inoculated rabbits [90]. This suggests that anti-Tp0136 antibodies may not be protective. When compared to Tp0136-immunized and unimmunized rabbits, Tp0663-immunized rabbits had a significantly lower treponemal load at the main lesion sites; and the chancre displayed in Tp0663-immunized rabbits healed more rapidly [79].

However, whether Tp0751 is a feasible immunization candidate is still up for debate. In a study done by Karen V. Lithgow, Tp0751 immunization was found to lessen the severity of lesions, slow the spread of *T. pallidum*, and cause more immune cells to gather at the sites of lesions. What’s exciting is that lymph nodes collected from Tp0751-immunized NZW rabbits failed to cause productive infection when injected into naive NZW rabbits [81]; on the other hand, Amit Luthra demonstrated that immunization with Tp0751, a bipartite *T. pallidum* lipoprotein with an intrinsically disordered region and lipocalin fold, fails to induce regulatory or protective antibodies in the rabbit model of experimental syphilis [91]. Future confirmation of the immunoprotective characteristics of Tp0751 might necessitate the use of novel approaches, such as genetic modification.

Other *T. pallidum* recombinant proteins have been shown to elicit powerful antibody or T-cell immune responses that suppress the progression of syphilitic lesions; these include Tp0126, Tp0821, TpGpd, TprI, Tp17, and the extracellular loop (ECL) of Tp0856 and Tp0858 [5, 82, 92–94]. While more research is needed to confirm that the aforementioned recombinant proteins indeed have immunoprotective properties, the data presented here should be useful in shaping future vaccine research.

## Preclinical models

**Fig. 2:**
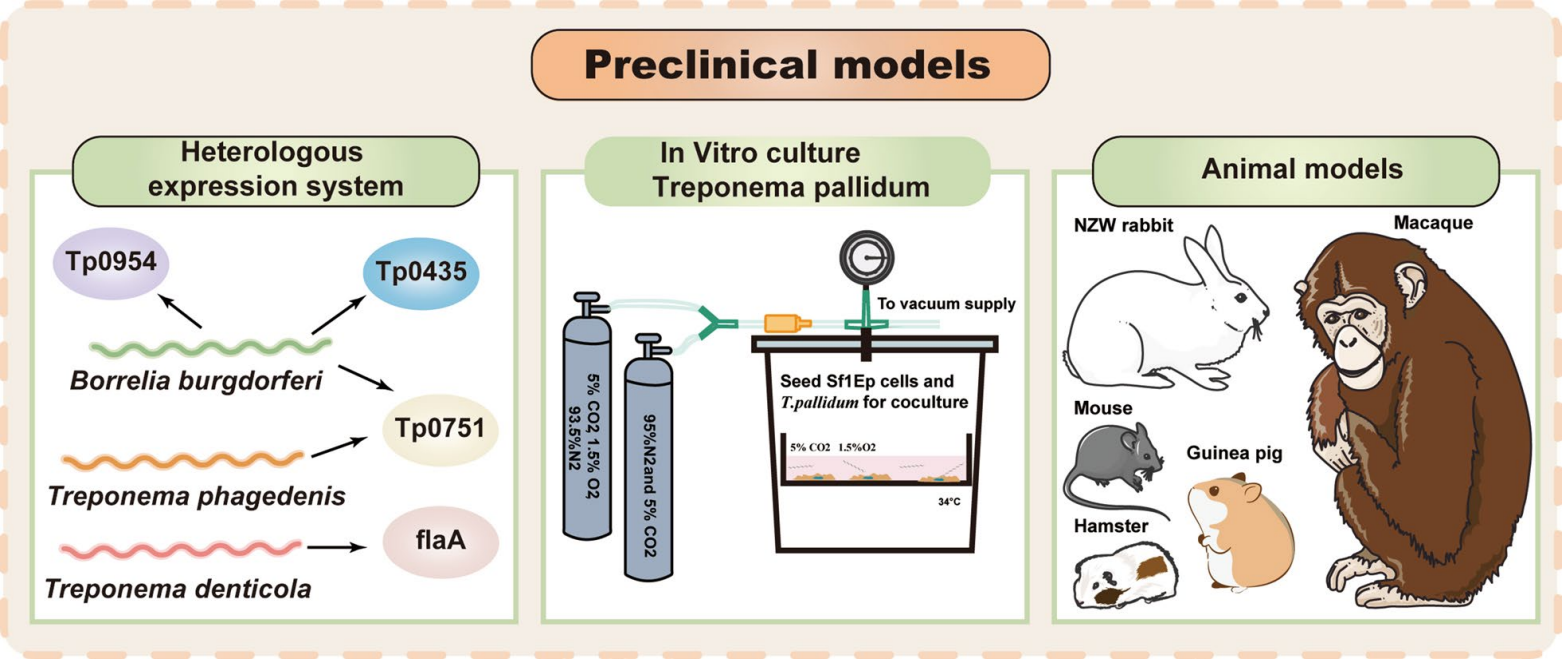
Preclinical models. At present, the commonly used preclinical syphilis models include heterologous expression models, in vitro culture models, and animal models. Heterologous expression models, such as *Borrelia burgdorferi*, *oral spirochete Treponema*, and *Treponema phagedenis*, contributed to identifying and determining the functional characteristics of *T. pallidum* proteins. Tp0435, Tp0954, and Tp0751 were identified as adhesins by a *Borrelia burgdorferi* heterologous expression system and adhered to mammalian endothelial cells and placental cell lines. The first long-term in vitro culture of *T. pallidum* was reported in 2018. This system utilized coincubation of the *T. pallidum* with Sf1Ep cottontail rabbit epithelial cells in a microaerobic environment containing 1.5% oxygen and 5% CO_2_. An altered medium, TpCM-2, is very important; the principal modification was the replacement of the basal medium, Eagle’s minimal essential medium (Eagle’s MEM), with a more complex tissue culture medium, CMRL 1066. Successful models of *T. pallidum* infection have been established in a variety of animals, including the NZW rabbit, nonhuman primate (NHP) (macaque), LSH hamster, guinea pig, and mouse. In light of its convenience and inexpensive cost, the NZW rabbit model of *T. pallidum* infection is favored by scientists. Although *T. pallidum* can successfully infect mice, it lacks obvious clinical manifestations. As a result, further study is required before mice may be used as a model for syphilis research. Preclinical models are utilized in studies on the underlying features and pathophysiology of *T. pallidum* infection, and they may be exploited to produce cutting-edge diagnostics and vaccines

Despite the availability and evidence-based treatment option of penicillin for more than 70 years, syphilis remains a public health threat. At the same time, due to practical and ethical considerations, research on therapeutic interventions and vaccine development for human subjects is limited. Therefore, relevant preclinical models are needed to investigate vaccine development and new treatments for syphilis. Preclinical models also play a key role in clinical transformation. The ability to conduct both forward translation, the process of translating basic research findings into practice, and reverse translation, the process of elucidating the mechanistic basis of clinical observation, will greatly improve the ability to develop effective anti-syphilis control strategies [95]. In addition to the routinely used cell lines and primary cell models (peripheral blood or bone marrow), a series of novel experimental models, such as in vitro culture and mouse models (Fig. 2), have recently emerged.

### Heterologous expression models

Before in vitro co-culture systems were developed, several heterologous expression models, such as *Borrelia burgdorferi*, *oral spirochete Treponema,* and *Treponema phagedenis*, contributed to identifying and determining the functional characteristics of *T. pallidum* proteins. The extracellular pathogen Borrelia burgdorferi, which can also cause systemic diseases, seems to be the best model. Borrelia burgdorferi serves as a model organism because both Tp0435 and Tp0954 have been shown to act as adhesins and adhere to mammalian ECs, gliomas, and placental cell lines [96, 97]. In addition, Tp0751, heterologously expressed by strains of *Borrelia burgdorferi,* not only mediates spirochete attachment to endothelial cells, but also plays a role as a vascular adhesin [98, 99]. Interestingly, a strong humoral response was observed by Parveen in NZW rabbits immunized against non-pathogenic *Borrelia burgdorferi* expressing TprK and was partially protective [82]. Further advancements in in vitro culture systems and genetic manipulation technologies may eventually replace the heterologous expression system of Borrelia burgdorferi, which has the potential to characterize the functional properties of *T. pallidum* proteins and virulence factors.

### In vitro culture models

*T. pallidum* is one of the few pathogenic bacteria that is notoriously challenging to culture in vitro, as is well-known. A system consisting of *T. pallidum* coculture with cottontail rabbit epithelium (Sf1Ep) was initially proposed in the early 1980s, but *T. pallidum* could only survive in vitro for 1 to 2 weeks [100]. Recently, Edmondson et al. have found that *T. pallidum* could be consistently cultured in a modified Sf1Ep co-culture system, in which continuous growth of *T. pallidum* in vitro was dependent upon co‐culture with Sf1Ep cottontail rabbit epithelial cells in a specialized tissue culture medium (*T. pallidum* culture medium 2, or TpCM‐2) under microaerobic conditions (a GasPak™ 150 vented anaerobic jar [Brewer jar] filling with 1.5% O_2_, 5% CO_2_, and 93.5% N_2_) ever since 2018 [52, 101]. *T. pallidum*’s remarkable in vitro culture system greatly aided in the creation of genetic tools. With the help of homologous recombination, Romeis et al. were able to replace the TprA (Tp0009) pseudogene in the SS14 *T. pallidum* strain with a kanamycin resistance (kanR) cassette [73]. This discovery will allow the application of functional genetics techniques to study syphilis pathogenesis and improve syphilis vaccine development.

### Animal models

In addition, scientists may learn more about syphilis's causes, cures, and prevention methods with the use of animal models. Successful models of *T. pallidum* infection have been established in a variety of animals, including the NZW rabbit, nonhuman primate (NHP) (macaque), LSH hamster, guinea pig, and mouse [102–105]. As far as we know, the NZW rabbit and the NHP model (macaques) are the only animals whose syphilis phenotypes (lesions) are most comparable to those seen in humans [102, 103]. In light of its convenience and inexpensive cost, the NZW rabbit model of *T. pallidum* infection is favored by scientists. For isolating novel strains of *T. pallidum* from clinical samples, the NZW rabbit model is also crucial [106]. Therefore, the rabbit model is not only the best animal model for studying syphilis vaccine candidates, but also the most widely used model by researchers, as it allows for in-depth pathogenesis research, evaluation of new therapies, and testing of potential vaccine candidates [81, 91]. Nevertheless, genetic modification of rabbit models is more challenging; as technology advances, CRISPR/Cas9 may help alleviate this difficulty. The first transgenic rabbit line was created in 1985, and in 2014, CRISPR/Cas9 was successfully used to create gene-knockout rabbits [107]. It is also worth noting that the present assembly of the rabbit genome is still incomplete. Off-target effects are a major problem with the Cas9 system for gene targeting. It is expected that the researchers will be able to produce viable models of immunodeficient and knockout rabbits for use in syphilis studies in the near future.

Despite the importance of rabbit models, their use is still limited compared to mice. Mice models have become powerful tools for many studies, in large part due to the lower variability between individuals, the lower cost, and the wide availability of reagents. A syphilitic infection model in C57BL/6 mice has been developed recently [105]. The study has shown that *T. pallidum* can colonize the heart, liver, spleen, kidney, testis, and brain of C57BL/6 mice after infection, but the inflammatory response of C57BL/6 mice after infection is mild and lacks obvious clinical manifestations [105]. As a result, further study is required before mice may be used as a model for syphilis research.

## Conclusion and outlook

Clinical strategies for the diagnosis, control, and prevention of *T. pallidum* have advanced in sophistication with the growing understanding of its pathogenic mechanisms. The diagnosis of syphilis is complex, and serological testing is still the gold standard for the diagnosis of syphilis patients. It is worth noting that NAAT may be a promising method for detecting *T. pallidum* DNA in syphilis patient samples. In addition to the routine diagnosis of syphilis by NAAT in blood and CSF, scholars have recently begun to experiment with biomarker and *T. pallidum* DNA testing in other specimen types as a proxy for transmissibility, including saliva, saliva, urine, semen, oropharynx, and anorectum. Recently, researchers have developed a PCR-LwCas13a syphilis assays that may offer a promising alternative to sequencing-based methods for molecular surveillance and drug resistance genotyping. As metabolomics and proteomics breakthroughs are made, biomarkers based on those technologies are also being found to be exciting. Meanwhile, vaccines for syphilis have recently been developed, mainly targeting outer membrane proteins and various membrane lipoproteins of *T. pallidum*. The Tpr paralogous homologous protein family, including TprK, TprL, TprC, and TprD, has also been extensively studied through genome sequencing and bioinformatics analysis of the most common strains in clinical practice, providing a favorable basis for future vaccine studies. In addition, with the development of *T. pallidum* in vitro culture and genetic modification of these preclinical models, it will further provide more rapid, accurate, and effective methods for the diagnosis, treatment, and prevention of syphilis.

## Data Availability

Not applicable.
